# Mapping the SLP76 interactome in T cells lacking each of the GRB2-family adaptors reveals molecular plasticity of the TCR signaling pathway

**DOI:** 10.3389/fimmu.2023.1139123

**Published:** 2023-03-15

**Authors:** Kilian Ruminski, Javier Celis-Gutierrez, Nicolas Jarmuzynski, Emilie Maturin, Stephane Audebert, Marie Malissen, Luc Camoin, Guillaume Voisinne, Bernard Malissen, Romain Roncagalli

**Affiliations:** ^1^ Centre d’Immunologie de Marseille-Luminy, Aix Marseille Université, INSERM, CNRS, Marseille, France; ^2^ Centre d’Immunophénomique, Aix Marseille Université, INSERM, CNRS UMR, Marseille, France; ^3^ Institut Paoli-Calmettes, CRCM, Aix Marseille Université, CNRS, INSERM, Marseille Protóomique, Marseille, France

**Keywords:** TCR, SLP76, GRB2-family adaptors, mass spectrometry, T cell signaling

## Abstract

The propagation and diversification of signals downstream of the T cell receptor (TCR) involve several adaptor proteins that control the assembly of multimolecular signaling complexes (signalosomes). The global characterization of changes in protein-protein interactions (PPI) following genetic perturbations is critical to understand the resulting phenotypes. Here, by combining genome editing techniques in T cells and interactomics studies based on affinity purification coupled to mass spectrometry (AP-MS) analysis, we determined and quantified the molecular reorganization of the SLP76 interactome resulting from the ablation of each of the three GRB2-family adaptors. Our data showed that the absence of GADS or GRB2 induces a major remodeling of the PPI network associated with SLP76 following TCR engagement. Unexpectedly, this PPI network rewiring minimally affects proximal molecular events of the TCR signaling pathway. Nevertheless, during prolonged TCR stimulation, GRB2- and GADS-deficient cells displayed a reduced level of activation and cytokine secretion capacity. Using the canonical SLP76 signalosome, this analysis highlights the plasticity of PPI networks and their reorganization following specific genetic perturbations.

## Introduction

Engagement of the T cell receptor (TCR) by a peptide/MHC ligand triggers the coordinated activities of intracellular signaling pathways leading to T cell activation (for review (1),). Downstream of the TCR, intracellular signals are elicited and transmitted by specialized proteins with unique molecular properties. Among them, adaptors are defined by their lack of enzymatic activity and by their ability to bridge proteins together. This function is mediated by specific domains and motifs defining the molecular specificity of each adaptor. In T cells, SLP76 (also known as LCP2) is a cytosolic adaptor that through its constitutive association with GRAP2 (also known as GADS) interacts with the LAT transmembrane adaptor upon TCR stimulation (2, 3). Along with the SLP76-GADS complex, LAT has also the unique ability to recruit the two other GRB2-family adaptors, GRB2 and GRAP, which allow potentiation and diversification of proximal TCR signals (4, 5). Although these three adaptors own a conserved structure consisting of a central SH2 domain surrounded by two SH3 domains, it has been shown that they recruit different elements of the TCR signaling network defining their specificity. For example, earlier studies showed that GADS is required to induce SLP76 association with LAT while GBR2 promotes Ras/MAPK signaling by bridging the guanine exchange factor SOS to LAT (6). Although GRAP has also been found in LAT and SLP76 complexes, its exact role in T cell signaling remains unclear (7, 8). Previous studies also indicated that proper T cell activation requires cooperative interactions between several members of the LAT-SLP76 signalosomes leading to the formation of large multiprotein complexes (9–12). For example, multipoint binding facilitates recruitment to the LAT-SLP76 complex of the ITK and PLCγ1 effectors which are involved in calcium release (13–16). TCR signal diversification is also enhanced by the phosphorylation of transmembrane molecules which provide additional docking sites for adaptors localized near the priming module. For example, such a mechanism has been documented with CD6 that nucleates a LAT-independent SLP76 signalosome regulating T cell activation (8, 17, 18).

In contrast to the profound defects in T cell development exhibited by mice deficient in LAT or SLP76, milder defects have been observed in GADS, GRAP and GRB2 knockout mice, raising questions on their exact role within the TCR signaling pathway (19–23). These results likely highlight important properties of PPI networks subjected to perturbations, such as plasticity mediated by binding cooperativities or molecular redundancy and compensation effects.

The combination of affinity purification (AP) and Mass Spectrometry (MS) analysis is a robust and sensitive approach to identify protein-protein interactions (PPI) in a hypothesis-free fashion. Recent studies using this method have shown that it also permits to quantify interaction stoichiometries and monitor changes of PPI following receptor stimulation under near-physiological conditions (24–26). In parallel, the CRISPR (clustered, regularly interspaced, short palindromic repeats)/Cas9 (CRISPR associated protein 9) system has become a method of choice for editing genes of interest and assessing the resulting impacts at the cellular and organismal levels. Here, by combining genome editing techniques and AP-MS analysis we determined and quantified the molecular reorganization of the SLP76 interactome resulting from the inactivation of each of the three GRB2-family adaptors. Our results revealed a reorganization of the molecular components of the SLP76 signalosome following TCR stimulation. Although deletion of GADS or GRB2 had a significant impact on composition of the SLP76 interactome, it minimally affected proximal and early TCR signaling events. The molecular plasticity we observed in the SLP76 PPI was, however, sufficient to quantitatively reduce effector T cell functions.

## Results

### A cellular model to assess the impact of GRB2-family adaptor deficiencies

**Figure 1:**
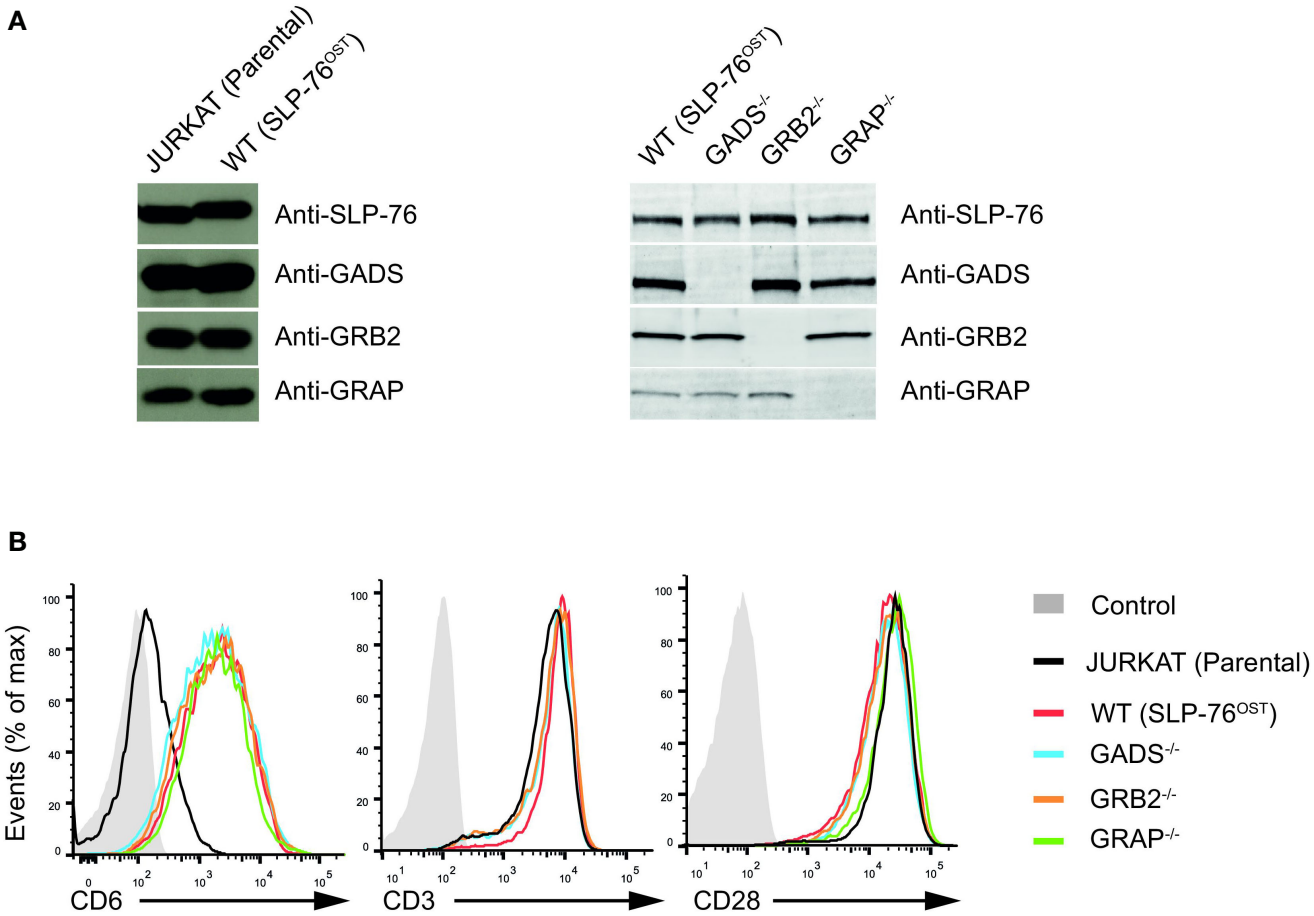
Generation and validation SLP76^OST^ Jurkat cells deficient in GRB2-family adaptors. **(A)** Immunoblot analysis of equal amounts of lysates from parental Jurkat, SLP76^OST^ (WT), SLP76^OST^GADS^-/-^ (GADS^-/-^), SLP76^OST^GRB2^-/-^ (GRB2^-/-^) and SLP76^OST^GRAP^-/-^ (GRAP^-/-^) cells probed with the indicated antibodies. **(B)** Expression of CD6, CD3 and CD28 at the surface of parental and Jurkat variants indicated in **(A)**, analyzed using flow cytometry. Data are representative of at least three independent experiments.

To examine the contribution of each of the GRB2-family adaptors to signal propagation and diversification by the SLP76/LAT signalosome, we performed a series of genetic modifications in the Jurkat cell line. Using the CRISPR/Cas9 system, we first introduced a OneStrepTag (OST) coding-sequence at the C-terminus of the two alleles of the SLP76 (*Lcp2*) gene. This modification allowed to generate a Jurkat cell line that expresses endogenous levels of SLP76^OST^ molecules (denoted here as wild-type (WT) Jurkat), permitting to analyze Jurkat cells with the AP-MS method already described in our previous studies (8, 24) (**Figure 1A**). Using the SLP76^OST^ Jurkat line, we subsequently derived Jurkat variants deficient in either GADS, GRB2 or GRAP by applying an additional step of CRISPR/Cas9 genome editing, targeting each of the three GRB2-family members (see Materials and Methods and **Supplementary Figure 1** for methodological details). Analysis of total lysates of the three variants by immunoblot with antibodies specific for GADS, GRB2 or GRAP (denoted as GADS^-/-^, GRB2^-/-^ or GRAP^-/-^ respectively) showed no residual expression of the targeted GRB2-family member (**Figure 1A**).

Jurkat cells express very low amount of CD6 on their surface. As CD6 is an important element of the SLP76 signalosome (8), we further introduced a cDNA encoding human CD6 by classical electroporation into the various Jurkat variants previously generated and selected transfectants expressing similar amounts of CD6 (**Figure 1B**). Following this last modification, we ensured that each variant had comparable surface levels of both CD3 and CD28 (**Figure 1B**). Considering that the sequential rounds on gene editing might impact on the global abundance or specific expression of critical proteins involved in the TCR signaling, we performed a total MS –based proteome for each of the Jurkat variants. Comparison of the proteomes of Jurkat variants with that of parental Jurkat cell line showed similar expression of major effectors involved in T cell activation. We also confirmed the presence of similar amounts of CD6 proteins and the absence of the targeted GRB2-family adaptor in the corresponding variant cell line (**Supplementary Figure 2** and **Supplementary Table 1**). Furthermore, and with the exception of GRAP that remains undetectable in proteomic analyses of the Jurkat variants, we were able to estimate that the cellular abundances per cell of SLP76 (around 3.5x10^4^ copies), GADS (around 1.3x10^5^ copies) and GRB2 (around 3.6x10^5^ copies) in Jurkat cells were similar to those in primary mouse CD4^+^ T cells (24, 27). Therefore, the series of CD6^+^ Jurkat cell lines expressing SLP76^OST^ molecules and deficient in each of the GRB2-family adaptors are suitable for both quantitative interactomics and functional analysis.

### The quantitative SLP76 interactome in Jurkat cells

**Figure 2:**
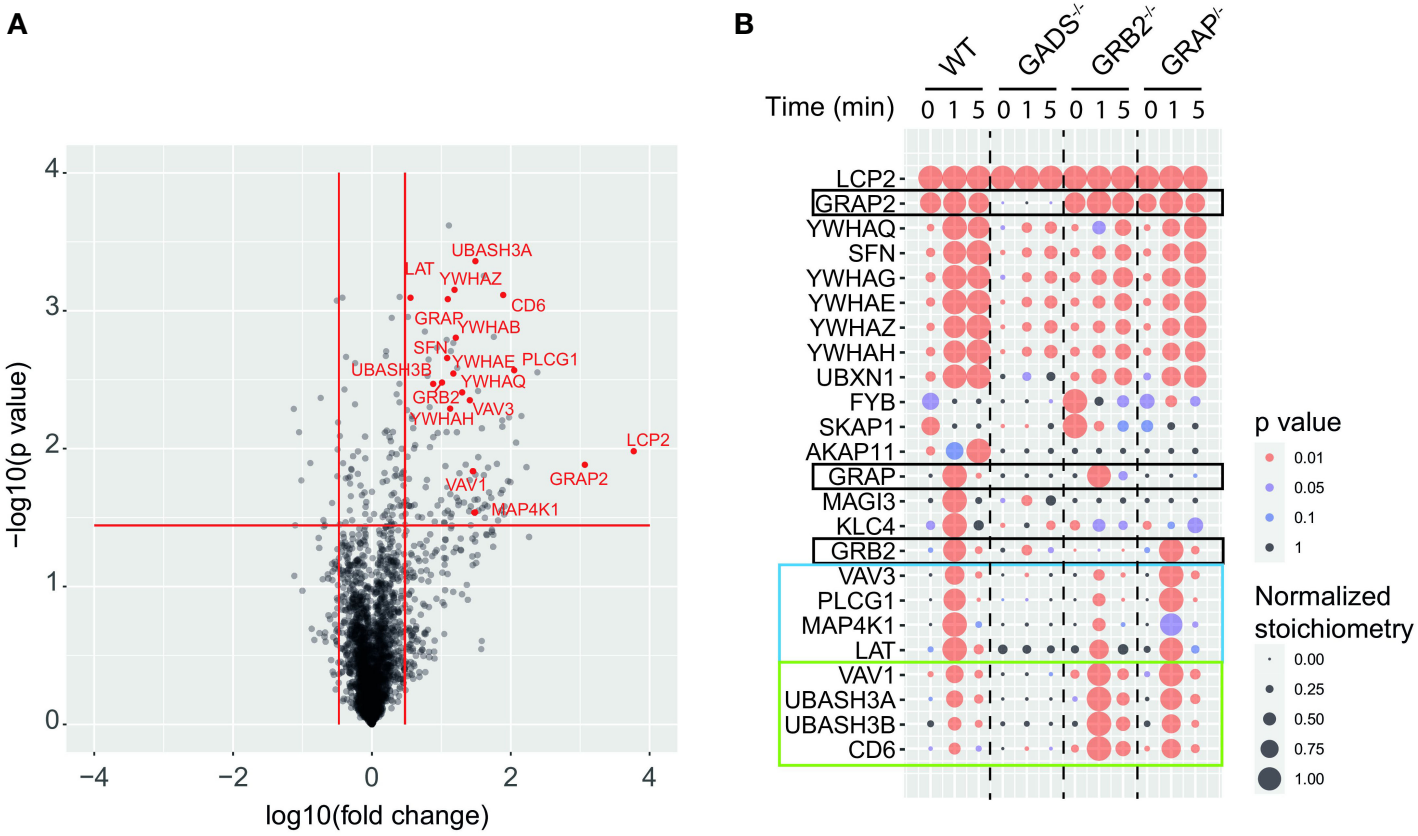
GRB2-family adaptors dependent and independent SLP76 protein interactions. **(A)** Volcano plot showing protein enrichment of affinity purified SLP76^OST^ from WT Jurkat cells compared to parental Jurkat cells expressing similar levels of untagged SLP76 proteins at 60 s after CD3 stimulation. Enriched preys are identified by a fold change > 3 and pvalue <0.01. Canonical SLP76 interactors are highlighted in red. **(B)** Dot plot showing the interaction stoichiometry of SLP76 with the 23 high-confidence preys for which the interaction stoichiometry is regulated by the inactivation of one the GRB2-family adaptors (fold change > 2.5 and pvalue <0.05). The interaction stoichiometry has been row-normalized to its maximum value observed over stimulation conditions (see key for Normalized Stoichiometry). The pvalue key is related to protein enrichment of the analysis in **(A)**. GRB2-family adaptors are framed in black. The two distinct SLP76 modules are framed in blue and green.

To determine how the absence of any one of the GRB2-family adaptors affected the SLP76 interactome, we stimulated WT and Jurkat variants with anti-CD3 for 1 or 5 minutes and purified SLP76^OST^ associated protein complexes using Strep-Tactin-Sepharose beads. After elution with D-biotin, protein complexes were identified and quantified by liquid chromatography coupled tandem MS (LC-MS/MS) analysis (see Materials and Methods). To determine proteins that were significantly associated with SLP76, we calculated the enrichment and corresponding p-value of log-transformed and normalized intensities of each detected protein between all SLP76^OST^ variants and the parental Jurkat expressing the OST-untagged SLP76 molecules. We determined that 131 proteins were significantly associated indirectly or directly with SLP76 (enrichment equal or greater than 3 and a p-value below or equal to 0.01) in at least one of the experimental conditions tested (**Figure 2A** and **Supplementary Table 2**). Global analysis of the SLP76 interactome in Jurkat indicated that it is very similar to that identified in mouse CD4^+^ T cells performed under similar conditions (24). For example, known components of the SLP76 signalosome such as PLCγ1, LAT, MAP4K1 (HPK1), CD6, VAV1/3, ITK and the 14-3-3 chaperones interacted with SLP76 upon TCR stimulation (**Figure 2A** and **Supplementary Table 2**). In addition to those canonical SLP76 interactors, the SLP76 interactome also contained UBASH3A and its relative UBASH3B exhibiting phosphatase activities and bearing an ubiquitin binding capacity. Consistent with previous findings and based on the interaction stoichiometry (**Supplementary Table 2**), our analysis showed that 100% of the SLP76 molecules are constitutively associated with GADS. In contrast, GRB2 and GRAP were detected with SLP76 only upon TCR engagement and with a much weaker interaction stoichiometry (approximatively 1% and 0.1% of SLP76 molecules bind to GRB2 and GRAP respectively). Although this was consistent with the low affinity binding capacity of GRB2 and GRAP to SLP76 (28, 29), these low stoichiometries probably more likely reflect the binding of GRB2 and GRAP to LAT molecules complexed to SLP76 (**Supplementary Figure 3** and **Supplementary Table 2**).

### Quantitative analysis of molecular reorganization induced by the absence of GRB2-family adaptors

Having determined the SLP76 interactome in WT Jurkat cells, we then analyzed the molecular rearrangements resulting from gene inactivation of each of the GRB2-family adaptors. Although conventional biochemical methods of protein detection can assess the loss or gain of PPIs for predetermined molecules, they cannot provide precise quantification of the molecular perturbations associated with a signaling network. These limitations can be circumvented by exploiting data from quantitative AP-MS to calculate and measure changes of interaction stoichiometry for each prey in the absence of GADS, GRB2 or GRAP. Accordingly, we identified protein interactions whose stoichiometries were significantly affected (fold change >2.5 with p-value <0.05) in variant Jurkat cells as compared to WT Jurkat cells in a least one experimental condition (**Figure 2B**). In that respect, analysis of the SLP76 interactome in the GADS deficient context showed a strong reduction in interaction stoichiometries of canonical SLP76 preys, upon TCR stimulation. Thus, peptides corresponding to LAT, PLCγ1, MAP4K1, CD6 and VAV1/3 were not significantly enriched compared to control samples indicating that these proteins were unable to bind SLP76 in the absence of GADS. Moreover, although strongly reduced, some residual interactions of SLP76 with 14-3-3 family proteins and GRB2 remained detectable upon TCR stimulation. A reduction expected since SLP76 bind to LAT *via* the GADS intermediate. Importantly, our results show that neither GRB2, nor GRAP can substitute for GADS in the TCR induced docking of SLP76 to LAT.

In contrast to GADS deficiency which has a major negative effect on the binding of most TCR-inducible SLP76 interactors, GRB2 deficiency resulted into two distinct patterns of interaction stoichiometry changes. The group of interactors composed of LAT, PLCγ1, VAV3, MAP4K1 and 14-3-3 family members showed a decrease SLP76 binding in absence of GRB2 whereas the binding of the CD6, UBASH3A/B and VAV1 interactors to SLP76 showed unaffected or higher interaction stoichiometries than in WT cells. These results were consistent with the participation of SLP76 to the LAT and CD6 signalosomes that form coincidently and independently following TCR triggering (8, 18). Indeed, they suggested that GRB2 can control the balance of these two protein complexes by competing with or promoting the recruitment of SLP76.

In contrast to GADS and GRB2, AP-MS of SLP76^OST^ in the context of GRAP deficient cells showed no major modification of the canonical SLP76 interactors identified and quantified for the experimental conditions tested. These data suggested that GRAP is dispensable or that its absence can be fully compensated by GRB2 or GADS for the assembly of the early TCR-induced SLP76 signalosome. Altogether, our analysis showed that quantitative AP-MS used in combination with molecular perturbation conditions can rapidly identify the reorganization of a global PPI network based on interaction stoichiometry.

### Molecular events associated with the perturbed SLP76 signalosome

**Figure 3:**
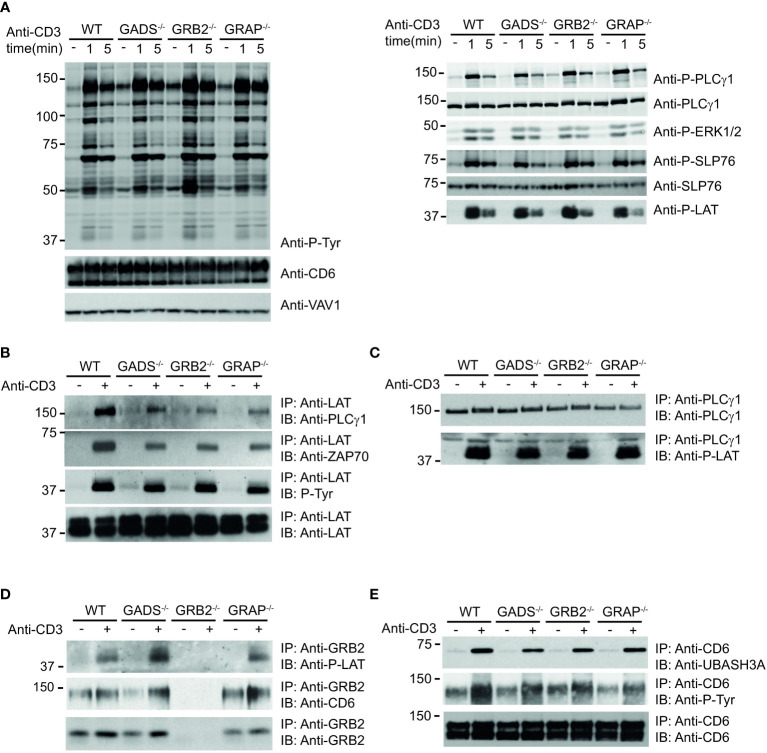
Analysis of TCR-induced molecular events in cells deficient in GRB2-family adaptors. **(A)** WT, GADS^-/-^, GRB2^-/-^ and GRAP^-/-^ Jurkat cells were left unstimulated (-) or stimulated for 1 or 5 min with anti-CD3 antibody. Equal amounts of protein lysates were analyzed by immunoblot with phospho-specific antibodies for tyrosine motifs (P-Tyr), ERK1/2 (T202/Y204), SLP76 (Y128), PLCγ1 (Y783) and LAT (Y191). VAV1 and total proteins serve as a loading control (left and right panel respectively). The CD6 expression was also assessed for each Jurkat variants. **(B–E)** The same cells as in **(A)** were left unstimulated (-) or stimulated (+) with anti-CD3 antibody and subsequently lysed. Equal amounts of cell lysates were incubated with the specified antibodies (IP) and the resulting immunoprecipitates analyzed by immunoblot with the antibodies specified in the left margin (IB). Data are representative of at least two independent experiments.

We next examined the impact resulting from each GRB2-family member deficiencies on TCR-induced signaling events. WT and Jurkat variants were either left untreated or stimulated with anti-CD3 antibody for 1 or 5 minutes and induced protein phosphorylation was assessed in whole cell lysates. Surprisingly, the global pattern of tyrosine phosphorylation triggered by TCR stimulation showed no major defect between WT and variant cells (**Figure 3A**). Moreover, further analysis with antibodies recognizing phospho-specific sites of PLCγ1 (Y783), LAT (Y171), SLP76 (Y128) and ERK1/2 (T202/Y204) showed a rather similar level of TCR-induced phosphorylation between all cell types.

Thus, although most PPIs involving SLP76 are highly impacted by the absence of GADS and GRB2, the phosphorylation of the molecules involved in the LAT-SLP76 complexes remain largely unaffected. Given that some of these phosphorylations mediate PPIs, we wanted to investigate whether other signaling complexes related to the LAT-SLP76 hub could still assemble. Immunoprecipitation of LAT confirmed its phosphorylation state and showed its preserved ability to bind PLCγ1 and ZAP70 in GRB2, GADS or GRAP deficient cells **(** **Figure 3B** **)**. Consistent with those data, a complementary examination of this complex with an immunoprecipitation of PLCγ1 showed no defect of its capacity to associate with LAT **(** **Figure 3C** **)**. To complete the analysis of the LAT signaling complex, we assessed its binding to GRB2. Immunoprecipitation of GRB2 showed that the LAT-GRB2 interaction was preserved in GADS and GRAP deficient cells **(** **Figure 3D** **)**.

CD6 is another transmembrane molecule that through its phosphorylated cytoplasmic tail recruits SLP76 (through GADS), GRB2, VAV and UBASH3 molecules (18). Purification and analysis of CD6 complexes showed normal CD6 phosphorylation and recruitment of the phosphatase UBASH3A upon TCR stimulation in wild-type cells and variants including GADS deficient Jurkat T cells **(** **Figure 3E** **)**. Moreover, GRB2 immunoprecipitation confirms the presence of SLP76/GADS and GRAP-independent CD6-GRB2 complexes **(** **Figure 3D** **)**. Altogether, these results indicated that some parts of the LAT and CD6 signalosomes can be assembled in the absence of any one of the GRB2-family adaptors. Given that SLP76 interactions with CD6 and LAT are lost in the absence of GADS (**Figure 1**), our results show that UBASH3A-CD6, GRB2-CD6, PLCγ1-LAT and GRB2-LAT interactions can arise in a SLP76 independent manner.

### Functional impacts of GRB2-family adaptors deficiencies

**Figure 4:**
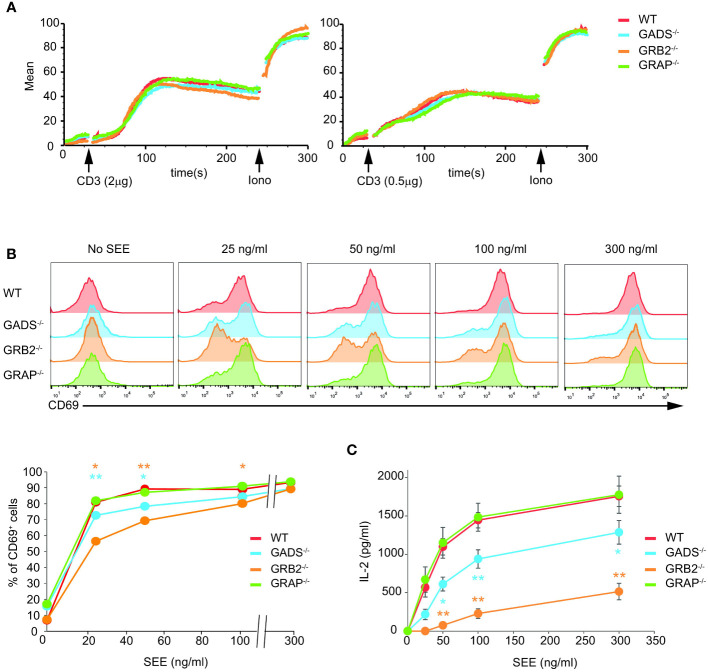
Impaired T cell functions in GADS and GRB2 deficient cells. **(A)** WT, GADS^-/-^, GRB2^-/-^ and GRAP^-/-^ Jurkat cells were loaded with Indo-1AM and stimulated with 2 or 0.5 μg of anti-CD3 antibody (final concentrations 5 μg/ml and 1.25 μg/ml respectively) and concentration of intracellular calcium was monitored for 5 min at 37°C by flow cytometry. The TCR-independent response was assessed by addition of Ionomycin (Iono) after 4 min. **(B)** WT and Jurkat variants were stimulated with Raji cells in presence of increasing amount of SEE. The expression of CD69 was assessed 24 h after stimulation by flow cytometry. A graph of percentage of CD69 positive cells was plotted as a function of SEE concentrations. **(C)** ELISA analysis of IL-2 production by the specified cells 24 h after stimulation with Raji cells and SEE. (mean +/- sd of technical replicates). Data are representative of at least three independent experiments.

We next assessed the cellular functions of Jurkat cells deficient for one of the GRB2-family adaptors. We first tested their capacity to mobilize calcium upon TCR stimulation by flow cytometry. Although we used weak doses of anti-CD3 antibody, we failed to detect significant defect in GADS, GRB2 or GRAP deficient cells (**Figure 4A**). To further examine cellular T cell functions, we co-incubated Raji lymphoblastoid B cells presenting the superantigen staphylococcal enterotoxin E (SEE) with Jurkat cells (**Figure 4B**). In this system, Jurkat cells secrete IL-2 and upregulate CD69 in a SEE dose dependent manner. GBR2 deficient cells showed a drastic reduction of CD69 expressing cells with low doses of SEE compared to WT cells (**Figure 4B**). A similar effect, although less pronounced, was observed with GADS deficient cells. Interestingly, we noticed that this defect was bypassed when high doses of SEE were used to stimulate Jurkat cells. In contrast, GRAP deficient cells showed normal induction of CD69. Then, we analyzed IL-2 secretion of WT and Jurkat variants under the same conditions of stimulation (**Figure 4C**). GRB2 and GADS deficient cells reproducibly secreted less IL-2 than WT and GRAP deficient cells. The use of increasing doses of SEE also indicated a stronger decrease of IL-2 production in the absence of GRB2 than GADS. Moreover, in contrast to the CD69 induction, the impaired IL-2 secretion remained apparent with high doses of SEE.

### Co-stimulatory signaling pathways remained unaffected by GRB2-family member deficiencies

**Figure 5:**
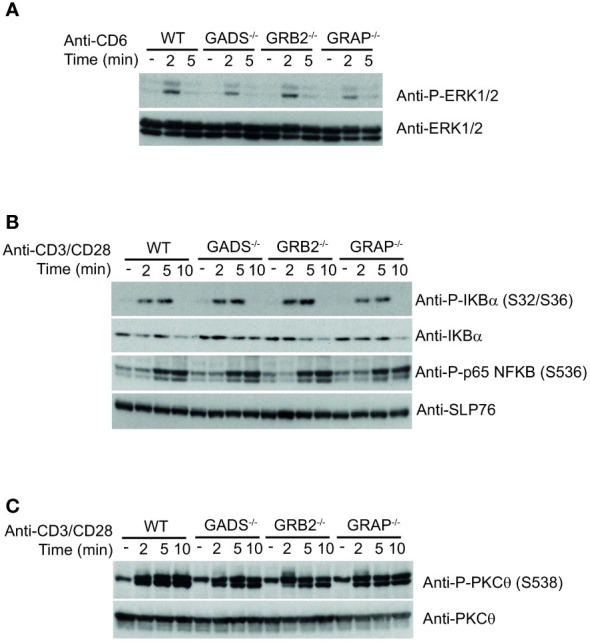
Normal co-stimulatory signaling in Jurkat cells deficient in GRB2-family adaptors. **(A)** WT, GADS^-/-^, GRB2^-/-^ and GRAP^-/-^ Jurkat cells were left unstimulated **(-)** or stimulated for 2 or 5 min with anti-CD6 antibody. Equal amounts of protein lysates were analyzed by immunoblot with phospho-specific ERK1/2 antibody and with anti-ERK1/2 antibody. **(B)** Same cells as in A were left unstimulated **(-)** or stimulated for 2, 5 or 10 min with anti-CD3 and anti-CD28 antibodies. Equal amounts of protein lysates were analyzed by immunoblot with indicated phospho-specific antibodies and loading control was assessed with anti-SLP76 antibody. **(C)** Analysis of the PKCθ phosphorylation in cells and experimental conditions described in **(B)**. Data are representative of at least two independent experiments.

The impaired IL-2 secretion and CD69 expression but normal ERK1/2 phosphorylation in GRB2 and GADS deficient cells suggested that other pathways than the TCR signaling pathway might be compromised by the absence of GRB2-family adaptors. It has been shown that CD6 engagement can promote ERK1/2 phosphorylation in a TCR-independent manner (8, 30). Given that GADS and GRB2 can directly associate with CD6 (18, 31), we investigated whether GBR2-family adaptors could be important for CD6-mediated ERK1/2 activation. Comparison of WT and variant Jurkat cells stimulated with anti-CD6 antibody showed no significant difference in their ability to promote ERK1/2 phosphorylation (**Figure 5A**). These results demonstrated that MAPK activation in this context, is independent of GBR2-family adaptors and possibly the SLP76 binding.

GRB2-family adaptors have also been associated with the CD28 signaling pathway. In particular, it has been suggested that GRB2 interaction with CD28 was important for IL-2 secretion (32, 33). To investigate the signaling defect associated with impaired functions of the GADS and GRB2 deficient cells, we sought to probe CD28-triggered NF-κB signaling. To trigger optimal activation of the NF-κB signaling pathway, we stimulated Jurkat cells with anti-CD3 plus anti-CD28 antibodies and analyzed phosphorylation events by immunoblotting. We observed that simultaneous engagement of the TCR and CD28 receptors induced effective phosphorylation of p65-NF-κB and IκBα and led to IκBα degradation in WT Jurkat cells (**Figure 5B**). In addition, the pattern of CD3-CD28 co-stimulation–induced NF-κB activation was similar between WT and cells deficient in any of the GRB2-family adaptors (**Figure 5B**). Consistently, TCR-CD28 stimulation led to normal phosphorylation of the kinase PKCθ, required for NF-κB activation (34, 35) (**Figure 5C**). Therefore, these results suggested that the activation of the NF-κB signaling pathway is fully preserved in the absence of GADS, GRB2 or GRAP.

### Preserved SLP-76-related molecular events in the absence of GRB2-family adaptors

**Figure 6:**
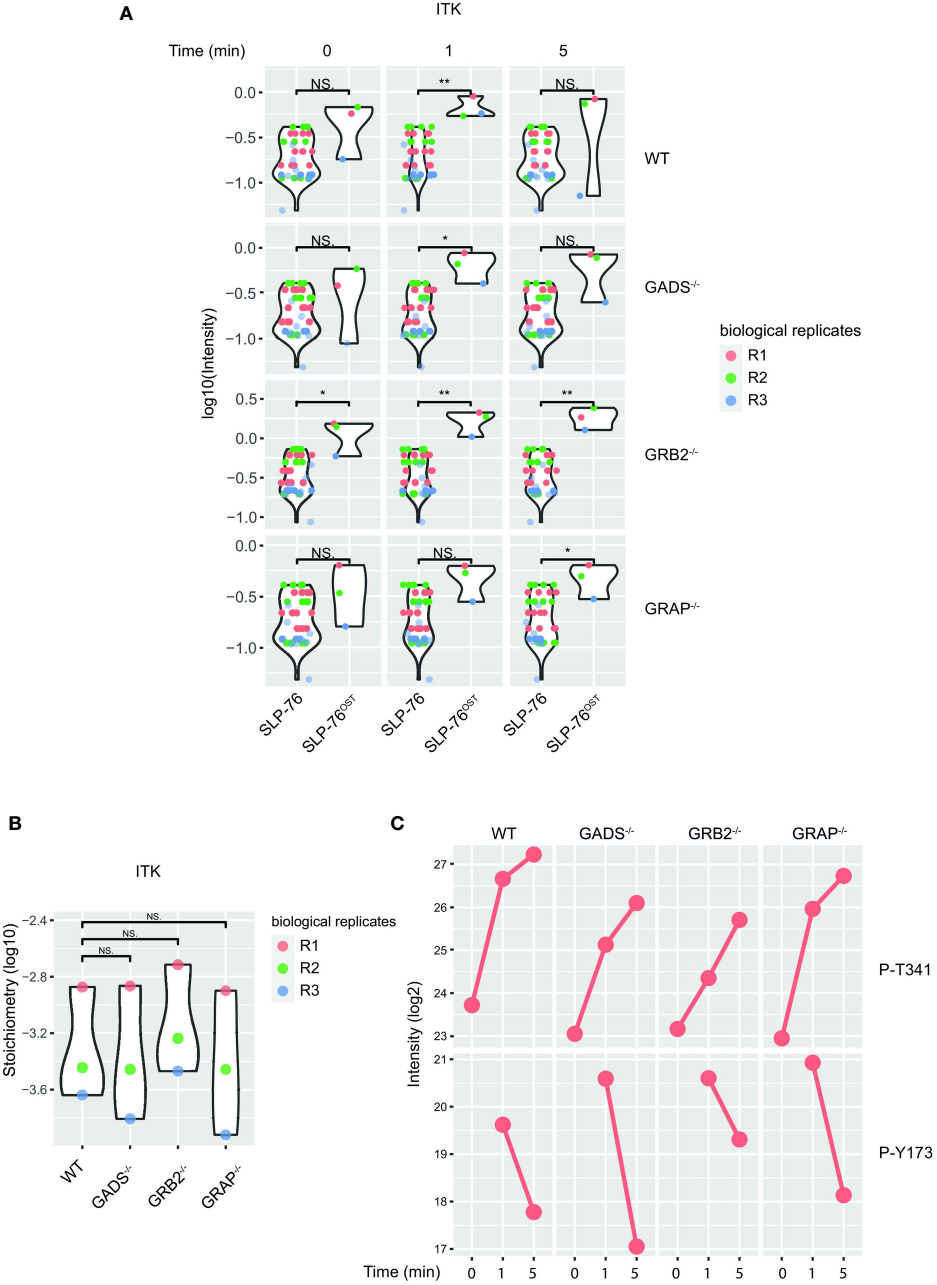
Preserved ITK-SLP76 interaction in the absence of GRB2-family adaptors. **(A)** The abundances of ITK was estimated in SLP76^OST^ AP-MS from cells that were left unstimulated (0) or stimulated for 1 or 5 min with anti-CD3 antibody in three independent biological replicates (R1, R2 and R3). **(B)** Comparison of ITK-SLP76 interaction stoichiometry in cells stimulated with anti-CD3 for 1 min. Imputed missing values are represented with lighter shaded dots. **(C)** MS intensities of specified phosphosites detected on the SLP76^OST^ molecules in experimental conditions described in **(A)** WT refers to the SLP76^OST^ variant while GADS^-/-^, GRB2^-/-^ and GRAP^-/-^ refer to the SLP76^OST^ variants deficient for GADS (GRAP2), GRB2 and GRAP respectively. Normalized intensities (see Materials and Methods) from parental Jurkat (SLP76) and SLP76^OST^ cells were compared using a two-sided Welch t-test (symbols used according to the t-test P-value: N.S., P > 0.05; *P ≤ 0.05; **P ≤ 0.01.

Despite a large dismantlement of the SLP76-nucleated complex, GADS deficient cells generate normal proximal TCR signals, suggesting that a small number of preserved SLP76-related molecular events are sufficient to induce T cell activation. Although our analysis of the SLP76^OST^ interactome indicated that some proteins such as GRB2 and 14-3-3 chaperones were still significantly recruited in the absence of GADS, their very weak interaction stoichiometries prevent a satisfying understanding of the phenotype observed (**Figure 2B**). To identify PPI preserved in the absence of any of the GRB2-family member, we applied an alternative filtering approach consisting in identifying enriched SLP76^OST^ PPIs for which stoichiometry is induced by TCR stimulation but remains unaffected in the various variants. Among the proteins matching these criteria, we found that the kinase ITK associated to the same extend with SLP76 in the absence of GADS, GRB2 or GRAP after TCR engagement (**Figures 6A, B**).

In addition to PPIs, another interesting aspect of the AP-MS approach is that it allows the identification of post-translational modifications of detected proteins, and in particular of the bait protein. In the case of the SLP76^OST^ interactome, the analysis of phospho-peptides showed that the phosphorylations of several residues of SLP76 were regulated upon TCR stimulation (**Figure 6C**). Among those, we consistently identified increased phosphorylation of the tyrosine 173 (Y173) in WT cells and in Jurkat cells deficient in any of the GRB2-family adaptors. This tyrosine residue has been shown to be phosphorylated by ITK, thereby allowing the priming and phosphorylation of PLCγ1. Together with the intact phosphorylation of PLCγ1 (**Figure 3**), these data suggested that in the absence of GADS, SLP76-mediated activation of ITK is preserved. Moreover, our data show that the SLP76-ITK interaction can occur independently of recruitment of SLP76 to the CD6 and LAT complexes.

## Discussion

Here, by combining the CRISPR/Cas9 gene editing system with AP-MS, we report an efficient method to quantitatively study changes in interactomes in living cells subjected to molecular perturbations. We used AP-MS to measure dynamic and stoichiometric changes in a PPI network and disentangle the composition of molecular complexes impacted by genetic mutations leading to protein loss. In particular, we analyzed the impact of the GRB2-family adaptors deficiency on the SLP76 signaling module and identified PPIs that are lost or preserved during TCR activation.

The absence of GADS severely compromised the formation of the SLP76 signalosome by preventing association with most of its canonical interactors. Although the loss of LAT interaction could have been anticipated, our data also indicated that SLP76 cannot properly associate with CD6 in such a context. This result is consistent with the reported bivalent GADS/SLP-76-CD6 binding model in which GADS plays a dominant role (31). The sensitivity of AP-MS made it possible to detect that TCR stimulation still enhanced association of the 14-3-3 chaperones and GRB2 with SLP76 in the absence of GADS. However, the SLP76-GRB2 interaction stoichiometry was significantly reduced (more than 2.5 folds) indicating that only a very small fraction of SLP76 was associated with GRB2 in this condition. Considering that the GRAP-SLP76 interaction was completely lost, these results suggest limited competition of the GRB2-family adaptors for SLP76 binding sites and imply that GRB2 and GRAP associations with SLP76 are mainly indirect and occur within the LAT and CD6 complexes. From a complementary point of view, this result indicates that, although reduced, some SLP76-nucleated complexes can still assemble outside of the LAT and CD6 signalosomes. This view is further supported by the TCR-induced ITK-SLP76 interaction and SLP76 phosphorylations that remained fully preserved in a system where GADS was absent. In particular, it has been shown that SLP76 Y173 is a substrate of ITK and its phosphorylation is required for PLCγ1 priming (36). Our analyses failed to detect significant differences in PLCγ1 phosphorylation and calcium release in cells deficient in GRB2-family adaptors. Consistently, GADS deficiency did not prevent LAT-PLCγ1 interaction and phosphorylation. Previous studies using cells deficient or knockdown in GADS have reported some defects in PLCγ1 phosphorylation and calcium mobilization (19, 37–39). Several reasons could account for these differences. Primary T cells from GADS-deficient mice showed impaired T cell development in the thymus which could explain stronger signaling abnormalities in the periphery. In Jurkat cells, the addition of CD6, which is weakly expressed in the parental cell line, could partially rescue the defect in calcium release that has been observed under suboptimal TCR stimulation conditions. While taking these considerations into account, these data suggest that ITK-dependent SLP76-mediated signaling on PLCγ1 can occur in LAT-independent manner and can eventually precede the LAT-PLCγ1 interaction.

The interaction stoichiometry analysis revealed an unexpected molecular reorganization of the SLP76 signalosome in GRB2 deficient cells. We observed a displacement of SLP76 toward the CD6 signaling module characterized by the presence of UBASH3 molecules (18), with a parallel reduction of interactions with LAT and PLCγ1. This result is consistent with the existence of two independent SLP76 pools generating different intracellular signals (8, 38, 40). We also observed that the absence of GRB2 induced opposite changes in the interaction stoichiometry of VAV1 and VAV3. This could be the result either of competition for SLP76 binding sites or of a distinct association mechanism of VAV molecules to SLP76. In both cases, these results suggest that the VAV molecules contribute to the specificity of the molecular composition and function of SLP76 pools. Numerous studies have demonstrated that VAV1 is involved in the TCR-induced calcium mobilization and in the cytoskeleton reorganization (41, 42). In contrast, the role of VAV3 in T cells is less documented, although previous studies have indicated that it appears to be disconnected from calcium release (43, 44). The emergence of several SLP76 pools and their specific molecular composition provides new and unexplored directions to understand the functions of VAV3 in T cells, particularly in the context of CD6 signaling. The PPI changes resulting from the absence of GRB2 also gives clues to its contribution to the SLP76 signalosome. The increased SLP76/GADS-CD6 interaction may suggest that GRB2 competes for the same CD6 docking sites. Alternatively, the absence of GRB2 leading to the destabilization of the SLP76/GADS-LAT complex, could increase the free SLP76/GADS pool that becomes available for association with uncomplete CD6 signaling modules. As GRB2 has been shown to mediate oligomerization and thus stabilization of the LAT signalosome (9), and because we have not noticed a significant increase in GBR2-CD6 interaction in GADS deficient cells (**Figure 3**), the second mechanism seems more likely.

CD6 is constitutively expressed in primary T cells and is considered as a co-modulator of T cell activation according to the immune response being measured (45) and its ability to recruit negative (UBASH3) and positive (SLP76, VAVs) effectors (18). As the parental Jurkat cell line is CD6^low^, we reconstituted *via* transfection physiological levels of CD6 expression that match those observed in primary T cells (8). Although the deficiency of GADS and GRB2 modified the CD6-SLP76 interaction stoichiometry, we observed a normal recruitment of the UBASH3 molecules to CD6. This finding supports the existence of SLP76/GADS/VAV and UBASH3 independent CD6-associated complexes and suggests a decoupling of the related positive and negative signals. Thus, molecular events mediated by the preserved UBASH3-CD6 interactions in GADS and GRB2 deficient cells, may exacerbate the alteration of effector functions resulting from the disorganized SLP76 signalosome. Future complementary analyses would be important to characterize the exact nature of the signals delivered by the UBASH3 molecules in this context.

Another intriguing result came with the analysis of the ERK-MAP kinase pathway and its apparent unaltered state in the absence of each GRB2-family adaptors. The full ERK activation can be achieved through two main routes, one involving membrane localization of the GRB2-SOS complex, the other being triggered by the PLCγ1-induced DAG production leading to RasGRP1 activation (46). Prior studies using GRB2 deficient mouse or cell lines knockdown in GRB2 reported no defect in TCR-induced ERK phosphorylation (22, 47). These results were further supported by the analysis of the SOS1/2 deficient mouse in which ERK activation was also normal after TCR ligation (48). Our data confirms that GBR2 is not required for TCR-induced ERK activation. Moreover, this result can be extended to GADS and GRAP for which the deficient cells displayed a similar phenotype. One explanation of these observations is that RasGRP1 plays a dominant role in comparison of the GRB2-SOS complex for the activation of the ERK-MAP kinase pathway upon TCR stimulation. In fact, this concept is strengthened by the absence of defect related to calcium release and PKC phosphorylation, the latter being dependent DAG production.

Taken together, our data challenge the canonical proximal TCR signaling model, and point out the complexity and plasticity of a protein signaling network subject to molecular perturbations. Moreover, the analysis of the rewiring of the PPI network under perturbations provided clues to the structure of protein complexes and allowed to infer direct or indirect protein-protein relationships. Thus, although expression of SLP76 is absolutely required for proper T cell activation, our results suggest that at least a part of its scaffolding capacity can be compensated or bypassed by other molecular mechanisms. However, it is important to remember that Jurkat cells present some abnormalities related to their leukemic status and which provide them, among other features, their proliferative capacity. These include multiple deficiencies in PTEN and SHIP-1, which result in deregulated cellular concentrations of phosphoinositides leading to the constitutive activation of the PI3K/AKT/mTOR signaling pathways (49, 50). Thus, it remains possible that GRB2-family members are involved in these pathways, a need that will be missed due to their constitutive activation in Jurkat cells. For example, the constitutive membrane localization of ITK in Jurkat cells could facilitate the recruitment and phosphorylation of effectors normally stabilized by GRB2-family members during TCR proximal signaling (49). These mechanisms could be sufficient to trigger essential early TCR signaling events but remained limited in their capacity to support long term activation, as highlighted by the altered T cell effector functions observed in GADS or GRB2 deficient cells. In conclusion we developed a comprehensively applicable workflow that could be implemented to analyze the molecular consequences of loss-of-function variants associated with immuno-deficiencies responsible of severe diseases.

## Materials and methods

### Cell lines

The Jurkat human leukemic cell line and Raji lymphoblastoid B cell line was provided by A. Weiss (University of California San Francisco, CA) and originated from American Type Culture Collection.

### CRISPR-Cas9–based genome editing of Jurkat T cells

We used a fast-track approach that permits to introduce biallelic null mutations in independent clones of Jurkat T cells (51). Briefly, sgRNA-specifying oligonucleotide sequences were chosen to minimize the likelihood of off-target cleavage based on publicly available on-line tools (https://www.dna20.com/products). The following pairs of sgRNAspecifying oligonucleotide sequences have been used:

tccagcttcacgttacagca to target exon 2 of GRAP2 (GADS)

gcactgagcagcgctcagaa to target exon 2 of GRB2

tggagccccgagcagcggca and agcagcggcatggagtccgt to target exon 1 of GRAP

The oligonucleotide guides also contained cacc and aacc overhangs for cloning into BbsI sites of plasmid pX330 (pSpCas9; Addgene plasmid ID 42230) or pX458 (pSpCas9(BB)-2A-GFP; Addgene plasmid ID 48138), and a G-C base pair was added at the 5’ end of the guide sequence for T7 transcription. To obtain in a single round of electroporation large numbers of independent Jurkat T cell clones with bi-allelic inactivation of GRAP2, GRB2 or GRAP, we devised a gene trapping strategy in which a neomycin resistance gene (NeoR)-containing cassette - called ‘bi-allelic KO type 1 cassette’ - is inserted in frame in exon 1 (GRAP) or 2 (GRAP2, GRB2) to lead to an early interruption of their open reading frame. **Supplementary Figure 1**, illustrates the strategy used for the GRAP gene. A similar strategy was used for the GRB2 and GRAP genes.

Insertion of the OST tag at the C-terminus of the SLP76 gene using the following pairs of sgRNA specifying oligonucleotide sequences have been used: caccgctcggctataacttgctat and caccggcacattaacgcatgctgc. The guide oligos contained overhangs for ligation into BbsI sites of plasmids pX330 and pX335 and a G-C base pair was added at the 5′ end of the guide sequence for T7 transcription. The annealed oligonucleotides were cloned into plasmid pX330 (pSpCas9; Addgene plasmid ID 42230) or into plasmid pX335 (hSpCas9n D10A; Addgene plasmid ID 42335). Double-stranded DNA repair templates (targeting vector) containing the desired edited sequence, as well as additional homologous sequences immediately upstream and downstream (homology arms) of the edited sequence, were assembled. The size of the left and right homology arms of the targeting vector was 500 bp. To prevent CRISPR-Cas9 cleavage of the targeting vectors, a silent mutation destroying the PAM sequence that is present in the genomic DNA was introduced into them. Finally, a LoxP-flanked NeoR cassette was introduced in the intron that flanked the exon subjected to editing. Jurkat, Clone E6-1 cells were nucleofected using the Cell Line Nucleofector kit V program I-010 for Nucleofector II with 2.5 μg of linearized targeting vector, and 10 μg pX330-sgRNA or pX335-sgRNA plasmid. Cells were allowed to recover for 48 h, and then subjected to G418 selection (2 mg/ml). Cells growing in G418 were then cloned by limiting dilution and screened for proper gene editing using PCR and genomic DNA sequencing. Transient expression of a Cre recombinase in correctly recombined Jurkat T cell clones allowed for the removal of the floxed NeoR cassette. Therefore, in addition to the intended mutation, each mutated allele contains a LoxP site in the 5′ or 3′ intron flanking the exon harboring the intended mutation. Clones with monoallelic mutation were defined as having PCR amplification of both the WT and mutated bands. Clones with biallelic mutation were defined as having PCR amplification of the mutated band and absence of the WT band. If a biallelic mutation was not obtained after a single round of nucleofection, clones lacking insertion-deletion (indel) in the untouched allele were subjected first to transient Cre expression to remove the NeoR cassette present in the properly targeted allele, and then to a second round of nucleofection using the same targeting vector and pX330-sgRNA plasmid.

### Flow cytometry

Stained cells were analyzed using an LSRII system (BD Biosciences). Data were analyzed with the Diva software (BD Biosciences). Cell viability was evaluated using SYTOX Blue (Life Technologies). The following antibodies were used: anti-CD3e (OKT3), anti-CD28 (CD28.2), anti-CD6(M-T605) and anti-CD69 (FN50); all from BD Biosciences.

### Jurkat T cell stimulation for functional assays

Jurkat T cells were stimulated by co-culture with Raji cells (0.5 × 10^5^) presenting the superantigen SEE (Toxin Technology), which binds to both the TCR and MHC class II molecules. IL-2 production by Jurkat cells was assessed 24 h after stimulation and was measured by ELISA (R&D Systems).

### Stimulation of Jurkat cells for interactomic analysis

40x10^6^ of wild-type or Jurkat cells expressing modified proteins with OST were left unstimulated or stimulated for 1 and 5 min with anti-CD3 antibody (UTHC1) at 37°C. Stimulation was stopped by the addition of lysis buffer (100 mM Tris, pH 7.5, 270 mM NaCl, 1 mM EDTA, 20% glycerol and 0.2% n-dodecyl-β-maltoside) supplemented with protease and phosphatase inhibitors. After 10 min of incubation on ice, cell lysates were centrifuged at 20,000g for 5 min at 4°C. Postnuclear lysates were incubated with StrepTactin beads for 1.5hrs at 4°C. Beads were washes five times with lysis buffer and associated protein complexes were subsequently eluted by addition of D-Biotin (2.5 mM). Protein extracts were loaded on NuPAGE 4–12% bis-Tris acrylamide gels (Life Technologies) to stack proteins in a single band that was stained with Imperial Blue (Thermo Fisher Scientific) and cut from the gel. Gels pieces were submitted to an in-gel trypsin digestion (Shevchenko et al., 1996). Peptides were extracted from the gel and dried under vacuum. Samples were reconstituted with 0.1% trifluoroacetic acid in 4% acetonitrile and analyzed by liquid chromatography (LC)-tandem MS (MS/MS) using a Q Exactive Plus Hybrid Quadrupole-Orbitrap online with a nanoLC Ultimate 3000 chromatography system (Thermo Fisher Scientific™, San Jose, CA). For each biological sample, 5 microliters corresponding to 33% of digested sample were injected in duplicate on the system. After pre-concentration and washing of the sample on a Acclaim PepMap 100 column (C18, 2 cm × 100 μm i.d. 100 A pore size, 5 μm particle size), peptides were separated on a LC EASY-Spray column (C18, 50 cm × 75 μm i.d., 100 A, 2 µm, 100A particle size) at a flow rate of 300 nL/min with a two steps linear gradient (2-22% acetonitrile/H20; 0.1% formic acid for 100 min and 22-32% acetonitrile/H20; 0.1% formic acid for 20 min). For peptides ionization in the EASYSpray source, spray voltage was set at 1.9 kV and the capillary temperature at 250°C. All samples were measured in a data dependent acquisition mode. Each run was preceded by a blank MS run in order to monitor system background. The peptide masses were measured in a survey full scan (scan range 375-1500 m/z, with 70 K FWHM resolution at m/z=400, target AGC value of 3.00×106 and maximum injection time of 100 ms). Following the high-resolution full scan in the Orbitrap, the 10 most intense data-dependent precursor ions were successively fragmented in HCD cell and measured in Orbitrap (normalized collision energy of 25%, activation time of 10 ms, target AGC value of 1.00×105, intensity threshold 1.00×104 maximum injection time 100 ms, isolation window 2 m/z, 17.5 K FWHM resolution, scan range 200 to 2000 m/z). Dynamic exclusion was implemented with a repeat count of 1 and exclusion duration of 20 s.

### Data analysis

Raw MS files were processed with MaxQuant software (v.1.6.3.4) for database search with the Andromeda search engine and quantitative analysis. Data were searched against the Homo sapiens entries of the UniProtKB protein database (release Swiss-Prot reviewed 2019_01, 20,412 entries), and the set of common contaminants provided by MaxQuant. Carbamidomethylation of cysteines was set as a fixed modification, whereas oxidation of methionine, protein N-terminal acetylation were set as variable modifications. Specificity of trypsin digestion was set for cleavage after K or R residues, and two missed trypsin cleavage sites were allowed. The precursor mass tolerance was set to 20 ppm for the first search and 4.5 ppm for the main Andromeda database search. The mass tolerance in tandem MS mode was set to 0.5 Da. Minimum peptide length was set to seven amino acids, and minimum number of unique or razor peptides was set to 1 for validation. The I = L option of MaxQuant was enabled to avoid erroneous assignation of undistinguishable peptides belonging to very homologous proteins. Andromeda results were validated by the target decoy approach using a reverse database, with a FDR value set at 1% at both peptide sequence match and protein level. For label-free relative quantification of the samples, the match between runs option of MaxQuant was enabled with a match time window of 1 min, to allow cross-assignment of MS features detected in the different runs, after alignment of the runs with a time window of 20 min. Protein quantification was based on unique and razor peptides. The minimum ratio count was set to 1 for label-free quantification calculation, and computation of the iBAQ metric was also enabled.

### Determination of cellular protein abundance in the proteome of Jurkat cells

Jurkat cells were washed twice with 1X PBS and cell pellets corresponding to 5 x 10^6^ cells were lysed in 150 ul of lysis buffer (Tris 50 mM, pH 7.5, EDTA 0.5 mM, NaCl 135 mM, SDS 1%) and kept on ice for 10 min. The samples were frozen and stored at -80°C before sample preparation for MS. 20 µg of proteins from each cell lysate were loaded and run on NuPAGE 4-12% bis-Tris acrylamide gels (Life Technologies) at 80V for only 8 minutes until the sample entered completely into the gel. Running was then stopped after this very short migration. In these conditions, proteins were stacked in a single band that can be stained with imperial blue, cut from the gel and after several washes, processed for reduction, alkylation and trypsin digestion. Protein identification and quantification was performed using liquid chromatography (LC)–tandem mass spectrometry (MS/MS) using an Orbitrap Fusion Lumos Tribrid mass spectrometer in-line with an Ultimate 3000 RSLCnano chromatography system (ThermoFisher Scientific). For each condition, 3 biological replicates were prepared and each replicate was injected twice on the LC-MS system. In a first step, peptides were concentrated and purified on a Dionex (C18 PepMap100, 2cm × 100µm I.D, 100Å pore size, 5µm particle size) pre-column using solvent A (0.1% formic acid in 2% acetonitrile). In a second step, peptides were separated on a reverse phase LC EASY-Spray C18 column from Dionex (PepMap RSLC C18, 50cm × 75µm I.D, 100Å pore size, 2µm particle size) at 300nL/min flow rate and 40° C. After equilibrating the column using 4% of solvent B (20% water - 80% acetonitrile - 0.1% formic acid), peptides were eluted from the analytical column by a two-step linear gradient (4-22% acetonitrile/H2O; 0.1% formic acid for 130 min and 22-32% acetonitrile/H2O; 0.1% formic acid for 15 min). For peptide ionization in the EASY-Spray nanosource, the spray voltage was set at 2.2kV and the capillary temperature at 275°C. The Advanced Peak Determination (APD) algorithm was used for real time determination of charges states and monoisotopic peaks in complex MS spectra. The mass spectrometer was used in data dependent mode to switch consistently between MS and MS/MS. Time between Masters Scans was set to 3 s. MS spectra were acquired in the 400-1600 m/z range at a FWHM resolution of 120 000 measured at 200 m/z. AGC target was set at 4.0.105 with a 50 ms maximum injection time. The most abundant precursor ions were selected and collision induced dissociation fragmentation at 35% was performed and analyzed in the ion trap using the “Inject Ions for All Available Parallelizable time” option with a dynamic maximum injection time. Charge state screening was enabled to include precursors with 2 and 7 charge states. Dynamic exclusion was enabled with a repeat count of 1 and a duration of 60 s.

### Data analysis of Jurkat cell proteome

Raw MS files were processed as previously described with MaxQuant software (v.1.6.3.4). Data were searched against the Homo sapiens entries of the UniProtKB protein database (release Swiss-Prot reviewed 2020_01, 20,367 entries). Protein entries from the MaxQuant ‘proteinGroups.txt’ output were first filtered to eliminate entries from reverse and contaminant databases. Cellular protein abundances were determined from raw intensities using the protein ruler methodology, using the following relationship: protein copies per cell = (protein MS signal × NA × DNA mass)/(M × histone MS signal), where NA is Avogadro’s constant, M is the molar mass of the protein and the DNA mass of a diploid human cell is estimated to be 6.5 pg. Cellular protein abundances were log transformed and averaged sequentially over technical and biological replicates.

### Statistical analyses related to MS

From the ‘proteinGroups.txt’ files generated by MaxQuant with the options described above, protein groups with negative identification scores were filtered, as well as proteins identified as contaminants. Protein intensities were log transformed before being normalized across all conditions (condition of stimulation, biological and technical replicates) by the median intensity. Normalized intensities corresponding to different technical replicates were averaged and missing values were replaced after estimating background binding from WT intensities. For each condition of stimulation, we used a two-tailed Welch’s t-test to compare normalized protein intensities detected in OST-tagged samples across all biological replicates and WT sample intensities pooled from all conditions of stimulation. Proteins were selected as specifically interacting with SLP76-OST when both the P-value was below P = 0.01 and the corresponding enrichment was equal or greater than three-fold.

## Data availability statement

The datasets presented in this study can be found in online repositories. The data presented in the study are deposited in the PRIDE repository, accession number PXD038891.

## Author contributions

KR performed most of the experiments with the help of JC-G, and EM. NJ performed CRISPR-Cas9 gene editing. SA and LC performed the MS experiments. MM provided key insights and GV the computational framework for MS analysis. RR and BM conceived the project. KR and RR wrote the manuscript with feedbacks from BM and GV. All authors contributed to the article and approved the submitted version.

## Supplementary material

Supplementary Figure 1 Schematic showing the inactivation of a GRAP allele following insertion of a ‘LoxP-Neo-LoxP-synthetic intron-synthetic PolyA’ cassette by homologous recombination. Jurkat cells with mono- or bi-allelic activation of GRAP can be readily selected in presence of G418.

Supplementary Figure 2 Proteomes of variant Jurkat T cell lines. Protein copy number comparisons between parental and variant Jurkat T cell lines. WT refers to the SLP76^OST^ variant while GADS^-/-^, GRB2^-/-^ and GRAP^-/-^ refer to the SLP76^OST^ variants deficient for GADS (GRAP2), GRB2 and GRAP respectively. The canonical effectors of the TCR signaling pathways are highlighted in red. Note that no specific GRAP peptides were detected in none of these proteomes.

Supplementary Figure 3 Dynamic of SLP76^OST^ interactions with GRB2-family adaptors. The abundances of GRB2-family adaptors and SLP76 were estimated for each time points and biological replicates (R1, R2 and R3). Normalized intensities (see Materials and Methods) from parental Jurkat (SLP76) and SLP76^OST^ cells were compared using a two-sided Welch t-test (symbols used according to the t-test P-value: N.S., P > 0.05; *P ≤ 0.05; **P ≤ 0.01; ***P ≤0.001). Imputed missing values are represented with lighter shaded dots.

Supplementary Table 1 List of the proteome of variant Jurkat cells expressed in copy numbers.

Supplementary Table 2 List of the SLP76–prey interactions identified in the present study. each line shows an interaction between SLP76 and a prey and includes the corresponding enrichments and stoichiometries before stimulation and at each stimulation time point.

## References

[1] Gaud G Lesourne R Love PE. Regulatory mechanisms in T cell receptor signalling. Nat Rev Immunol (2018) 18(8):485–97. doi: 10.1038/s41577-018-0020-8 29789755

[2] Jordan MS Koretzky GA. Coordination of receptor signaling in multiple hematopoietic cell lineages by the adaptor protein SLP-76. Cold Spring Harb Perspect Biol (2010) 2(4):a002501. doi: 10.1101/cshperspect.a002501 20452948 PMC2845197

[3] Yablonski D. Bridging the gap: Modulatory roles of the Grb2-family adaptor, gads, in cellular and allergic immune responses. Front Immunol (2019) 10:1704. doi: 10.3389/fimmu.2019.01704 31402911 PMC6669380

[4] Buday L Egan SE Rodriguez Viciana P Cantrell DA Downward J. A complex of Grb2 adaptor protein, sos exchange factor, and a 36-kDa membrane-bound tyrosine phosphoprotein is implicated in ras activation in T cells. J Biol Chem (1994) 269(12):9019–23. doi: 10.1016/S0021-9258(17)37070-9 7510700

[5] Balagopalan L Coussens NP Sherman E Samelson LE Sommers CL. The LAT story: a tale of cooperativity, coordination, and choreography. Cold Spring Harb Perspect Biol (2010) 2(8):a005512. doi: 10.1101/cshperspect.a005512 20610546 PMC2908767

[6] Horejsi V Zhang W Schraven B. Transmembrane adaptor proteins: Organizers of immunoreceptor signalling. Nat Rev Immunol (2004) 4(8):603–16. doi: 10.1038/nri1414 15286727

[7] Trüb T Frantz JD Miyazaki M Band H Shoelson SE. The role of a lymphoid-restricted, Grb2-like SH3-SH2-SH3 protein in T cell receptor signaling. J Biol Chem (1997) 272(2):894–902. doi: 10.1074/jbc.272.2.894 8995379

[8] Roncagalli R Hauri S Fiore F Liang Y Chen Z Sansoni A. Quantitative proteomics analysis of signalosome dynamics in primary T cells identifies the surface receptor CD6 as a lat adaptor-independent TCR signaling hub. Nat Immunol (2014) 15(4):384–92. doi: 10.1038/ni.2843 PMC4037560 24584089

[9] Houtman JC Yamaguchi H Barda-Saad M Braiman A Bowden B Appella E. Oligomerization of signaling complexes by the multipoint binding of GRB2 to both LAT and SOS1. Nat Struct Mol Biol (2006) 13(9):798–805. doi: 10.1038/nsmb1133 16906159

[10] Coussens NP Hayashi R Brown PH Balagopalan L Balbo A Akpan I. Multipoint binding of the SLP-76 SH2 domain to ADAP is critical for oligomerization of SLP-76 signaling complexes in stimulated T cells. Mol Cell Biol (2013) 33(21):4140–51. doi: 10.1128/MCB.00410-13 PMC3811887 23979596

[11] Lewis JB Scangarello FA Murphy JM Eidell KP Sodipo MO Ophir MJ. ADAP is an upstream regulator that precedes SLP-76 at sites of TCR engagement and stabilizes signaling microclusters. J Cell Sci (2018) 131(21). doi: 10.1242/jcs.215517 PMC6240300 30305305

[12] Su X Ditlev JA Hui E Xing W Banjade S Okrut J. Phase separation of signaling molecules promotes T cell receptor signal transduction. Science. (2016) 352(6285):595–9. doi: 10.1126/science.aad9964 PMC4892427 27056844

[13] Bogin Y Ainey C Beach D Yablonski D. SLP-76 mediates and maintains activation of the tec family kinase ITK *via* the T cell antigen receptor-induced association between SLP-76 and ITK. Proc Natl Acad Sci U S A. (2007) 104(16):6638–43. doi: 10.1073/pnas.0609771104 PMC1871838 17420479

[14] Beach D Gonen R Bogin Y Reischl IG Yablonski D. Dual role of SLP-76 in mediating T cell receptor-induced activation of phospholipase c-gamma1. J Biol Chem (2007) 282(5):2937–46. doi: 10.1074/jbc.M606697200 17148460

[15] Hallumi E Shalah R Lo WL Corso J Oz I Beach D. Itk promotes the integration of TCR and CD28 costimulation through its direct substrates SLP-76 and gads. J Immunol (2021) 206(10):2322–37. doi: 10.4049/jimmunol.2001053 PMC8113088 33931484

[16] Sukenik S Frushicheva MP Waknin-Lellouche C Hallumi E Ifrach T Shalah R. Dimerization of the adaptor gads facilitates antigen receptor signaling by promoting the cooperative binding of gads to the adaptor LAT. Sci Signal (2017) 10(498). doi: 10.1126/scisignal.aal1482 28951535

[17] Hassan NJ Simmonds SJ Clarkson NG Hanrahan S Puklavec MJ Bomb M. CD6 regulates T-cell responses through activation-dependent recruitment of the positive regulator SLP-76. Mol Cell Biol (2006) 26(17):6727–38. doi: 10.1128/MCB.00688-06 PMC1592849 16914752

[18] Mori D Grégoire C Voisinne G Celis-Gutierrez J Aussel R Girard L. The T cell CD6 receptor operates a multitask signalosome with opposite functions in T cell activation. J Exp Med (2021) 218(2). doi: 10.1084/jem.20201011 PMC7608068 33125054

[19] Yoder J Pham C Iizuka YM Kanagawa O Liu SK McGlade J. Requirement for the SLP-76 adaptor GADS in T cell development. Science. (2001) 291(5510):1987–91. doi: 10.1126/science.1057176 11239162

[20] Yablonski D Kuhne MR Kadlecek T Weiss A. Uncoupling of nonreceptor tyrosine kinases from PLC-gamma1 in an SLP-76-deficient T cell. Science. (1998) 281(5375):413–6. doi: 10.1126/science.281.5375.413 9665884

[21] Zhang W Sommers CL Burshtyn DN Stebbins CC DeJarnette JB Trible RP. Essential role of LAT in T cell development. Immunity. (1999) 10(3):323–32. doi: 10.1016/S1074-7613(00)80032-1 10204488

[22] Jang IK Zhang J Chiang YJ Kole HK Cronshaw DG Zou Y. Grb2 functions at the top of the T-cell antigen receptor-induced tyrosine kinase cascade to control thymic selection. Proc Natl Acad Sci U S A. (2010) 107(23):10620–5. doi: 10.1073/pnas.0905039107 PMC2890815 20498059

[23] Shen R Ouyang YB Qu CK Alonso A Sperzel L Mustelin T. Grap negatively regulates T-cell receptor-elicited lymphocyte proliferation and interleukin-2 induction. Mol Cell Biol (2002) 22(10):3230–6. doi: 10.1128/MCB.22.10.3230-3236.2002 PMC133801 11971956

[24] Voisinne G Kersse K Chaoui K Lu L Chaix J Zhang L. Quantitative interactomics in primary T cells unveils TCR signal diversification extent and dynamics. Nat Immunol (2019) 20(11):1530–41. doi: 10.1038/s41590-019-0489-8 PMC6859066 31591574

[25] Francavilla C Papetti M Rigbolt KT Pedersen AK Sigurdsson JO Cazzamali G. Multilayered proteomics reveals molecular switches dictating ligand-dependent EGFR trafficking. Nat Struct Mol Biol (2016) 23(6):608–18. doi: 10.1038/nsmb.3218 PMC7618131 27136326

[26] Caron E Roncagalli R Hase T Wolski WE Choi M Menoita MG. Precise temporal profiling of signaling complexes in primary cells using SWATH mass spectrometry. Cell Rep (2017) 18(13):3219–26. doi: 10.1016/j.celrep.2017.03.019 PMC5382234 28355572

[27] Howden AJM Hukelmann JL Brenes A Spinelli L Sinclair LV Lamond AI. Quantitative analysis of T cell proteomes and environmental sensors during T cell differentiation. Nat Immunol (2019) 20(11):1542–54. doi: 10.1038/s41590-019-0495-x PMC6859072 31591570

[28] Berry DM Nash P Liu SK Pawson T McGlade CJ. A high-affinity arg-X-X-Lys SH3 binding motif confers specificity for the interaction between gads and SLP-76 in T cell signaling. Curr Biol (2002) 12(15):1336–41. doi: 10.1016/S0960-9822(02)01038-2 12176364

[29] Seet BT Berry DM Maltzman JS Shabason J Raina M Koretzky GA. Efficient T-cell receptor signaling requires a high-affinity interaction between the gads c-SH3 domain and the SLP-76 RxxK motif. EMBO J (2007) 26(3):678–89. doi: 10.1038/sj.emboj.7601535 PMC1794392 17235283

[30] Ibanez A Sarrias MR Farnos M Gimferrer I Serra-Pages C Vives J. Mitogen-activated protein kinase pathway activation by the CD6 lymphocyte surface receptor. J Immunol (2006) 177(2):1152–9. doi: 10.4049/jimmunol.177.2.1152 16818773

[31] Breuning J Brown MH. T Cell costimulation by CD6 is dependent on bivalent binding of a GADS/SLP-76 complex. Mol Cell Biol (2017) 37(11). doi: 10.1128/MCB.00071-17 PMC5440646 28289074

[32] Harada Y Tokushima M Matsumoto Y Ogawa S Otsuka M Hayashi K. Critical requirement for the membrane-proximal cytosolic tyrosine residue for CD28-mediated costimulation in vivo. J Immunol (2001) 166(6):3797–803. doi: 10.4049/jimmunol.166.6.3797 11238622

[33] Okkenhaug K Wu L Garza KM La Rose J Khoo W Odermatt B. A point mutation in CD28 distinguishes proliferative signals from survival signals. Nat Immunol (2001) 2(4):325–32. doi: 10.1038/86327 11276203

[34] Sun Z Arendt CW Ellmeier W Schaeffer EM Sunshine MJ Gandhi L. PKC-theta is required for TCR-induced NF-kappaB activation in mature but not immature T lymphocytes. Nature. (2000) 404(6776):402–7. doi: 10.1038/35006090 10746729

[35] Wang X Chuang H-C Li J-P Tan T-H. Regulation of PKC-θ function by phosphorylation in T cell receptor signaling. Front Immunol (2012) 3. doi: 10.3389/fimmu.2012.00197 PMC3393885 22798961

[36] Sela M Bogin Y Beach D Oellerich T Lehne J Smith-Garvin JE. Sequential phosphorylation of SLP-76 at tyrosine 173 is required for activation of T and mast cells. EMBO J (2011) 30(15):3160–72. doi: 10.1038/emboj.2011.213 PMC3160187 21725281

[37] Lugassy J Corso J Beach D Petrik T Oellerich T Urlaub H. Modulation of TCR responsiveness by the Grb2-family adaptor, gads. Cell Signal (2015) 27(1):125–34. doi: 10.1016/j.cellsig.2014.10.005 25452106

[38] Bilal MY Zhang EY Dinkel B Hardy D Yankee TM Houtman JC. GADS is required for TCR-mediated calcium influx and cytokine release, but not cellular adhesion, in human T cells. Cell Signal (2015) 27(4):841–50. doi: 10.1016/j.cellsig.2015.01.012 PMC4339522 25636200

[39] Yankee TM Yun TJ Draves KE Ganesh K Bevan MJ Murali-Krishna K. The gads (GrpL) adaptor protein regulates T cell homeostasis. J Immunol (2004) 173(3):1711–20. doi: 10.4049/jimmunol.173.3.1711 15265900

[40] Hassan NJ Barclay AN Brown MH. Frontline: Optimal T cell activation requires the engagement of CD6 and CD166. Eur J Immunol (2004) 34(4):930–40. doi: 10.1002/eji.200424856 15048703

[41] Costello PS Walters AE Mee PJ Turner M Reynolds LF Prisco A. The rho-family GTP exchange factor vav is a critical transducer of T cell receptor signals to the calcium, ERK, and NF-kappaB pathways. Proc Natl Acad Sci U S A. (1999) 96(6):3035–40. doi: 10.1073/pnas.96.6.3035 PMC15890 10077632

[42] Fischer KD Kong YY Nishina H Tedford K Marengère LE Kozieradzki I. Vav is a regulator of cytoskeletal reorganization mediated by the T-cell receptor. Curr Biol (1998) 8(10):554–62. doi: 10.1016/S0960-9822(98)70224-6 9601639

[43] Zakaria S Gomez TS Savoy DN McAdam S Turner M Abraham RT. Differential regulation of TCR-mediated gene transcription by vav family members. J Exp Med (2004) 199(3):429–34. doi: 10.1084/jem.20031228 PMC2211790 14757747

[44] Charvet C Canonigo AJ Billadeau DD Altman A. Membrane localization and function of Vav3 in T cells depend on its association with the adapter SLP-76. J Biol Chem (2005) 280(15):15289–99. doi: 10.1074/jbc.M500275200 15708849

[45] Gonçalves CM Henriques SN Santos RF Carmo AM. CD6, a rheostat-type signalosome that tunes T cell activation. Front Immunol (2018) 9:2994. doi: 10.3389/fimmu.2018.02994 30619347 PMC6305463

[46] Roose JP Mollenauer M Ho M Kurosaki T Weiss A. Unusual interplay of two types of ras activators, RasGRP and SOS, establishes sensitive and robust ras activation in lymphocytes. Mol Cell Biol (2007) 27(7):2732–45. doi: 10.1128/MCB.01882-06 PMC1899892 17283063

[47] Warnecke N Poltorak M Kowtharapu BS Arndt B Stone JC Schraven B. TCR-mediated erk activation does not depend on sos and Grb2 in peripheral human T cells. EMBO Rep (2012) 13(4):386–91. doi: 10.1038/embor.2012.17 PMC3321150 22344067

[48] Guittard G Kortum RL Balagopalan L Çuburu N Nguyen P Sommers CL. Absence of both sos-1 and sos-2 in peripheral CD4(+) T cells leads to PI3K pathway activation and defects in migration. Eur J Immunol (2015) 45(8):2389–95. doi: 10.1002/eji.201445226 PMC4536155 25973715

[49] Shan X Czar MJ Bunnell SC Liu P Liu Y Schwartzberg PL. Deficiency of PTEN in jurkat T cells causes constitutive localization of itk to the plasma membrane and hyperresponsiveness to CD3 stimulation. Mol Cell Biol (2000) 20(18):6945–57. doi: 10.1128/MCB.20.18.6945-6957.2000 PMC88770 10958690

[50] Astoul E Edmunds C Cantrell DA Ward SG. PI 3-K and T-cell activation: limitations of T-leukemic cell lines as signaling models. Trends Immunol (2001) 22(9):490–6. doi: 10.1016/S1471-4906(01)01973-1 11525939

[51] Celis-Gutierrez J Blattmann P Zhai Y Jarmuzynski N Ruminski K Gregoire C. Quantitative interactomics in primary T cells provides a rationale for concomitant PD-1 and BTLA coinhibitor blockade in cancer immunotherapy. Cell Rep (2019) 27(11):3315–30.e7. doi: 10.1016/j.celrep.2019.05.041 31189114 PMC6581740

