# Supplementary figures and images for: Mapping the SLP76 interactome in T cells lacking each of the GRB2-family adaptors reveals molecular plasticity of the TCR signaling pathway
Source: Front Immunol. 2023 Mar 15;14:1139123. doi: 10.3389/fimmu.2023.1139123 (PMC10057548; doi:10.3389/fimmu.2023.1139123)

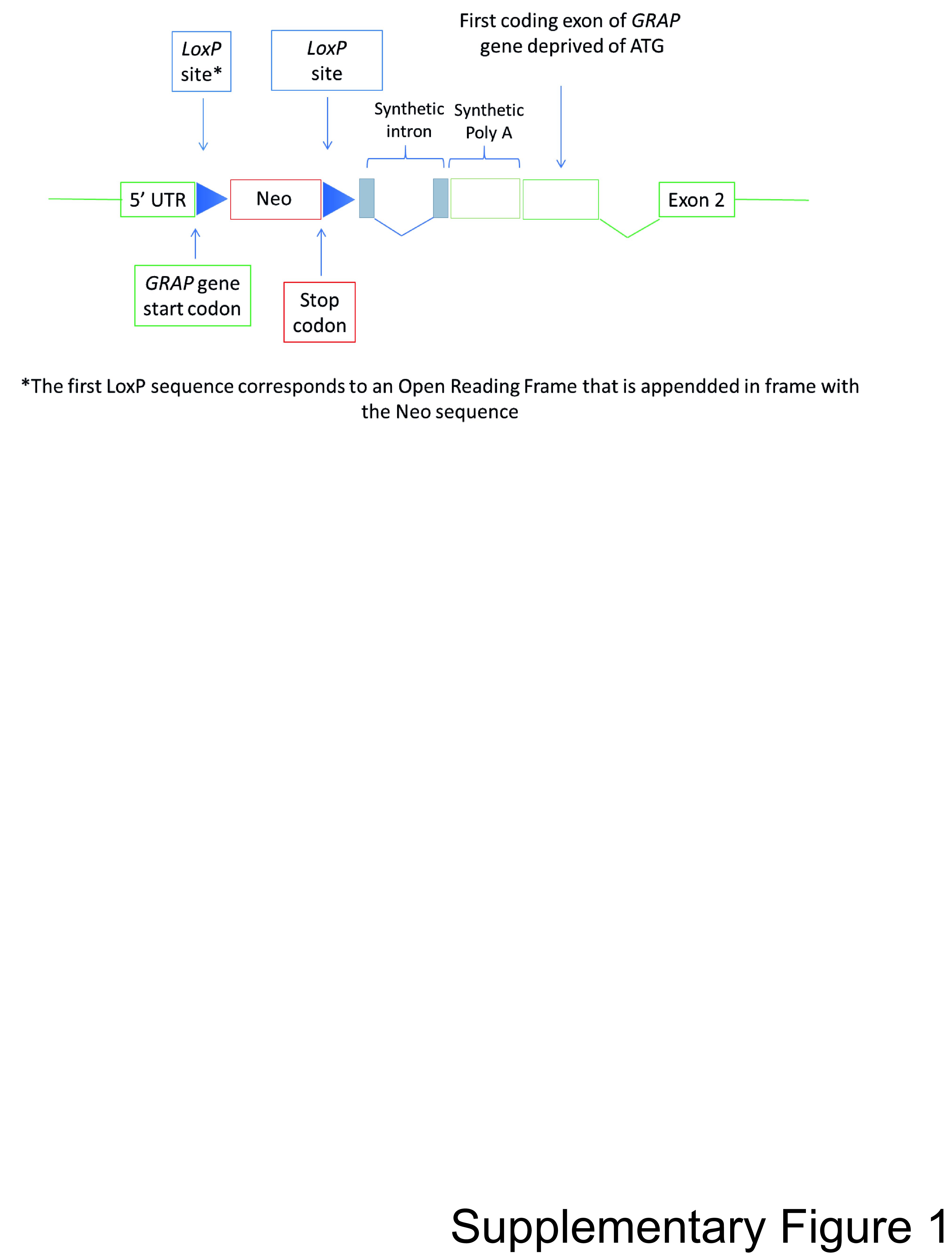

Supplement: Supplementary Figure 1 — Schematic showing the inactivation of a GRAP allele following insertion of a ‘LoxP-Neo-LoxP-synthetic intron-synthetic PolyA’ cassette by homologous recombination. Jurkat cells with mono- or bi-allelic activation of GRAP can be readily selected in presence of G418. [file Image_1.jpeg]

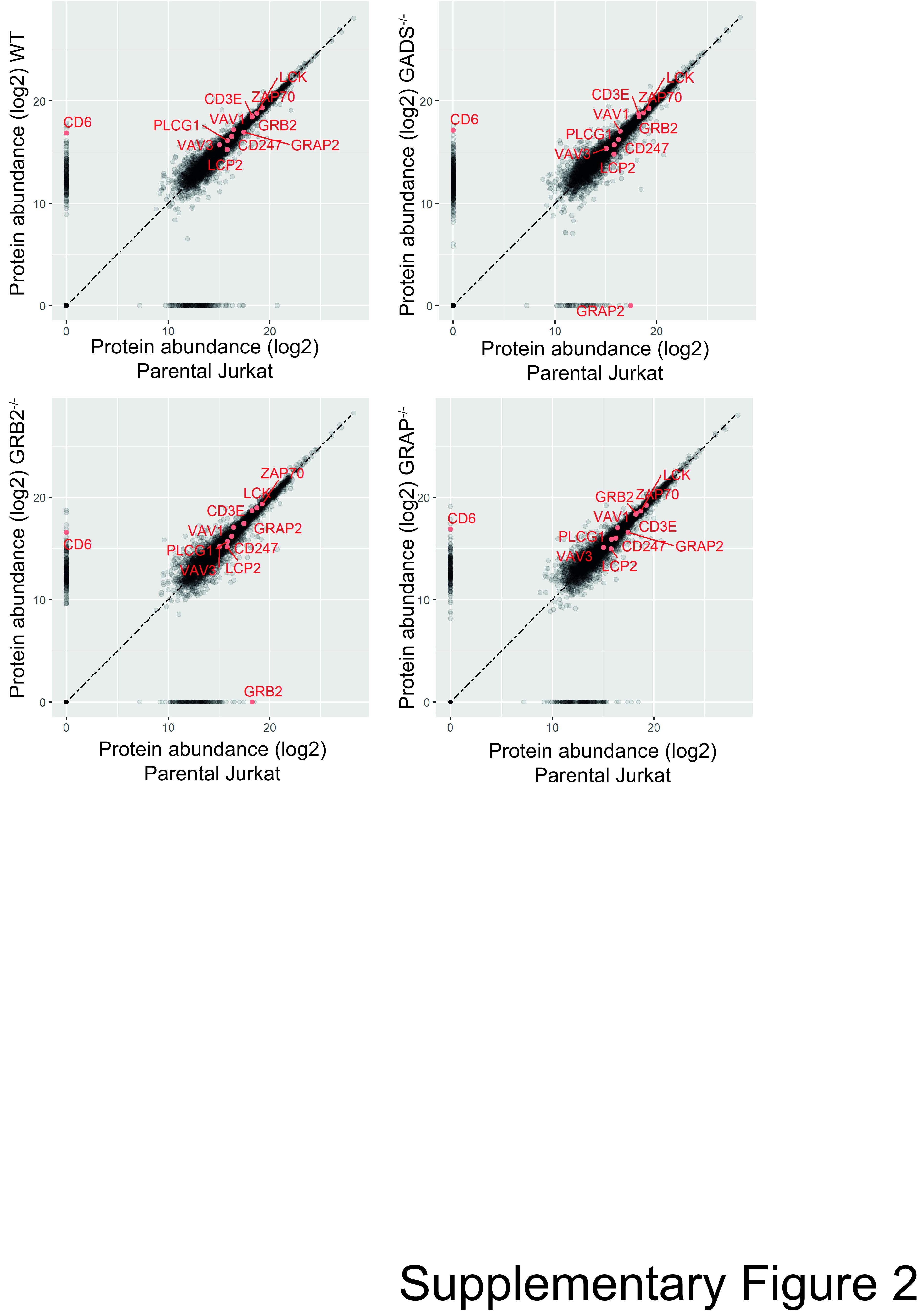

Supplement: Supplementary Figure 2 — Proteomes of variant Jurkat T cell lines. Protein copy number comparisons between parental and variant Jurkat T cell lines. WT refers to the SLP76OST variant while GADS-/-, GRB2-/- and GRAP-/- refer to the SLP76OST variants deficient for GADS (GRAP2), GRB2 and GRAP respectively. The canonical effectors of the TCR signaling pathways are highlighted in red. Note that no specific GRAP peptides were detected in none of these proteomes. [file Image_2.jpeg]

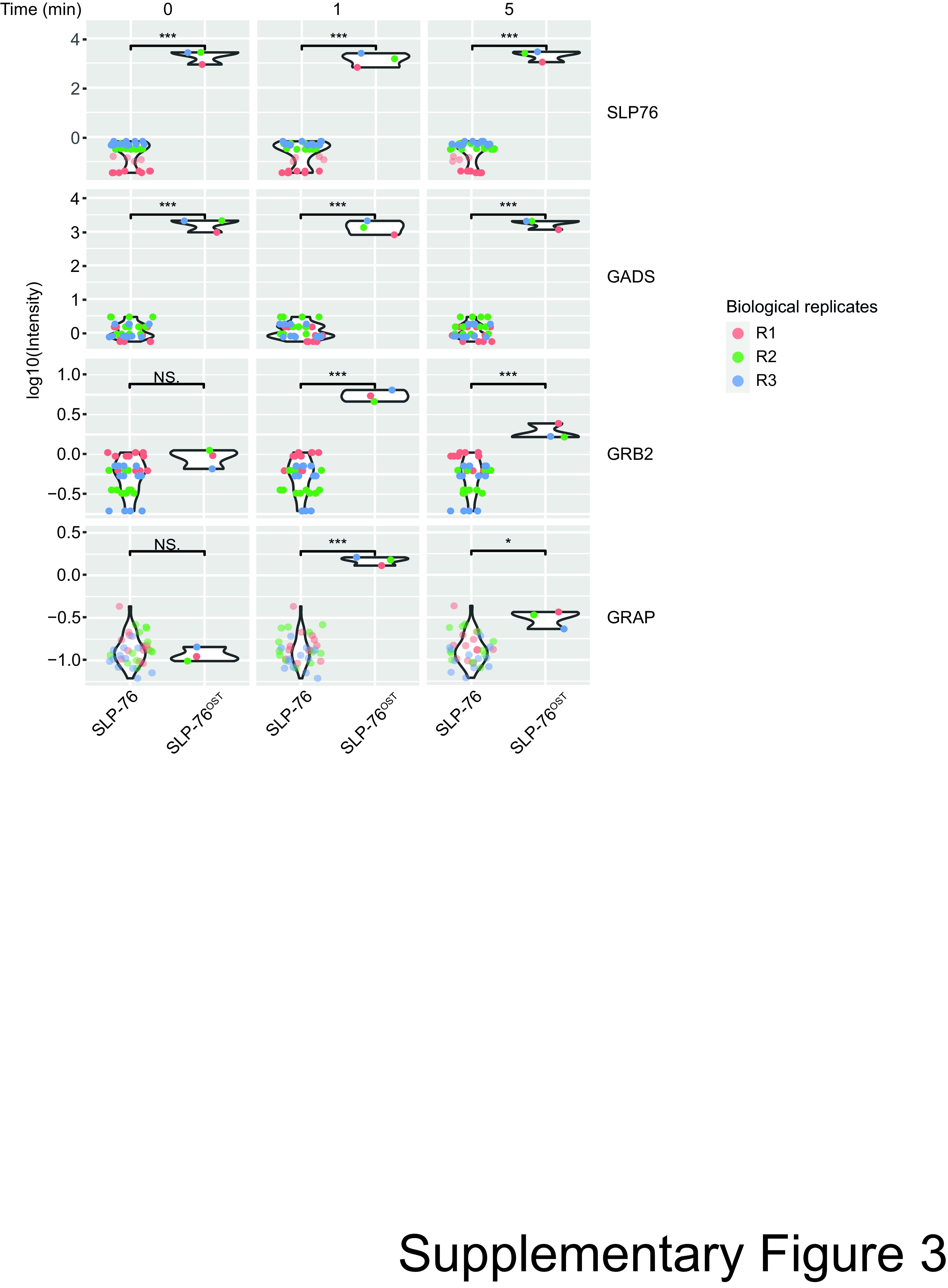

Supplement: Supplementary Figure 3 — Dynamic of SLP76OST interactions with GRB2-family adaptors. The abundances of GRB2-family adaptors and SLP76 were estimated for each time points and biological replicates (R1, R2 and R3). Normalized intensities (see Materials and Methods) from parental Jurkat (SLP76) and SLP76OST cells were compared using a two-sided Welch t-test (symbols used according to the t-test P-value: N.S., P > 0.05; *P ≤ 0.05; **P ≤ 0.01; ***P ≤0.001). Imputed missing values are represented with lighter shaded dots. [file Image_3.jpeg]
